# Plant Tissue Modelling Using Power-Law Filters

**DOI:** 10.3390/s22155659

**Published:** 2022-07-28

**Authors:** Samar I. Gadallah, Mohamed S. Ghoneim, Ahmed S. Elwakil, Lobna A. Said, Ahmed H. Madian, Ahmed G. Radwan

**Affiliations:** 1 Nanoelectronics Integrated Systems Center (NISC), Nile University, Giza 12588, Egypt; s.imbaby@nu.edu.eg (S.I.G.); lsaid@nu.edu.eg (L.A.S.); 2 Department of Electronics and Communication Engineering, Egypt University of Informatics, New Administrative Capital, Cairo 11578, Egypt; mohamed_ghoneim@ieee.org; 3 Department of Electrical and Computer Engineering, University of Sharjah, Sharjah P.O. Box 27272, United Arab Emirates; elwakil@ieee.org; 4 Department of Electrical and Software Engineering, University of Calgary, Calgary, AB T2N 1N4, Canada; 5 Radiation Engineering Department, National Centre for Radiation Research and Technology, Egyptian Atomic Energy Authority, Cairo 11787, Egypt; 6 Engineering Mathematics and Physics Department, Cairo University, Giza 12613, Egypt; agradwan@ieee.org; 7 School of Engineering and Applied Sciences, Nile University, Giza 12588, Egypt

**Keywords:** bio-impedance, power-law filters, fractional-order circuits, optimization

## Abstract

Impedance spectroscopy has became an essential non-invasive tool for quality assessment measurements of the biochemical and biophysical changes in plant tissues. The electrical behaviour of biological tissues can be captured by fitting its bio-impedance data to a suitable circuit model. This paper investigates the use of power-law filters in circuit modelling of bio-impedance. The proposed models are fitted to experimental data obtained from eight different fruit types using a meta-heuristic optimization method (the Water Cycle Algorithm (WCA)). Impedance measurements are obtained using a Biologic SP150 electrochemical station, and the percentage error between the actual impedance and the fitted models’ impedance are reported. It is found that a circuit model consisting of a combination of two second-order power-law low-pass filters shows the least fitting error.

## 1. Introduction

A plant is a living organism that consists of billions of cells organized as tissues, each having specific functions and purposes. However, certain components are common in all plant cells. For example, a cell stores its DNA in the nucleus, the main building block of a cell, embedded in the cytoplasm, while water and nutrients are stored in the vacuole. All components are surrounded by a plasma membrane that controls the transportation of materials into and out of the cell. Plant tissue condition has been used recently as a monitoring technique for studying the effects of various environmental conditions. However, in most studies this is performed by using invasive techniques that damage the tissues.

Meanwhile, bio-impedance spectroscopy measures the response of biological tissues under the effect of electrical current flow with varying frequency (AC signal). The impedance of a tissue can be correlated to its chemical and physical characteristics, which allows its assessment under changing environmental conditions [1,2]. Typically, the cellular membrane acts as a high impedance under the effect of a low-frequency AC signal. Thus, the current flows around the cells [3,4]. At higher signal frequencies, the cellular membrane acts as two capacitor plates allowing the current to flow through the cells and leading to lower impedance. The effects of many environmental conditions on plants have been investigated in several studies, such as fruit maturity in [5,6], ripening in [7,8] and bruising in [9].

Bio-impedance modelling was first successfully performed using the Cole-Cole impe- dance model (single dispersion model), which is one of the simplest models [10,11]. The Hayden model was proposed in 1969 to demonstrate and characterize the components inside a tissue cell but showed some defects in accurately fitting impedance data [12]. The double-shell model was introduced in 1990 to provide better fitting results while representing the cell-building components by taking the cell’s vacuole into consideration [1,4]. Despite that, the double-shell model did not provide efficient fitting results at low frequencies [13]. The Cole-Cole model was extended to the double dispersion impedance model to improve the accuracy over a wide range of frequencies [14,15]. By adding a Constant Phase Element (CPE) to the aforementioned models, better accuracy can be obtained. The CPE is a fractional-order capacitor with a fractional operator $\left(s^{\alpha }={\left(j\omega \right)}^{\alpha }\right)$. The Cole-Cole model was used for analyzing the effect of freezing and heating conditions on fruits [16].

More electrical models were used to characterize certain plants such as the Grout Citrus Fruit model, which was proposed to model constituent parts of the orange [17]. In [18], a bio-impedance model was proposed to track and analyze the natural dehydration dynamics and properties in onions. In [19], a Warburg impedance was used to represent the interface between the electrode and the surface of the fruit. Similar models were also proposed for modelling the electrode/tissue interface based in [20,21]. Plant stem was characterized through two fractional-order-based models in [15]. Fractional-order mathematical functions such as the Mittag-Leffler function were used in [22] to model the macroscale properties of complex and heterogeneous biological systems.

Regardless of which model is used, the model parameters are usually identified using meta-heuristic optimization techniques [3]. Deterministic algorithms such as the non-linear least square method were conventionally employed in bio-impedance parameter extraction problems but showed no robustness against noise [23] and tended to fail as the problem size grew larger [24]. Recently, meta-heuristic optimization algorithms were used to overcome these shortcomings. Meta-heuristic algorithms have aided in solving many optimization problems [25,26] and proved higher efficiency in the extraction of impedance models’ parameters [23]. Meta-heuristic algorithms are inspired by natural and biological activities such as water flow from rivers into sea [27], life habits of the red fox [28], mating behaviour of black widow spiders [29], decency behaviour of wild horses [30], and flight behaviour of Mayflies [31]. Different meta-heuristic optimization algorithms were compared in [26] for extracting bio-impedance models’ parameters. Some new algorithms such as the Flower pollination algorithm (FPA) and the Moth Flame optimization were also considered in [11]. The Water Cycle Algorithm (WCA) was deployed to extract equivalent plant stem model parameters in [15] and compared with other optimization algorithms.

Power-law filters were introduced recently in [32], where standard first-order or second-order filter transfer functions are raised to a fractional-order power α; i.e., $H_{f}\left(s\right)={\left[H_{m}\left(s\right)\right]}^{\alpha }$. Here, $H_{m}$ denotes the mother function of the standard filter. The methods efficient for approximating power-law filters are either frequency response curve fitting or padé approximations [33]. Single and double dispersion impedance models were represented by power-law filters in [34]. Recall that the Cole-Cole impedance model is expressed by:

(1) $$Z\left(s\right)=R_{\infty }+\frac{R_{c}}{\left(1+s^{\alpha }C_{\alpha }R_{c}\right)}=R_{\infty }+\frac{R_{c}}{\left(1+\frac{s^{\alpha }}{w_{o}}\right)},$$

where $R_{\infty }$ is a high frequency resistance, $R_{o}$ is a low frequency resistance, and $R_{c}$ is the difference between $R_{o}$ and $R_{\infty }$. The normalized impedance $Z_{n}\left(S\right)$ is expressed as:

(2) $$Z_{n}\left(s\right)=\frac{Z(s)}{R_{\infty }}=1+\frac{k}{\left(1+\frac{s^{\alpha }}{\omega_{o}}\right)},$$

where $k=R_{c}/R_{\infty }$, thus, according to the previous expression, the Cole model could be visualized as a fractional-order low pass filter with gain k. This filter can incorporate the power-law concept by raising the normalized impedance to a fractional-order power. It is worth noting that some approximations for modelling power-law filters were proposed in [35,36] to provide improved accuracy and stability but with higher computational complexity. The use of power-law filters in bio-impedance modelling has two advantages [34], namely (i) flexibility in selecting the number of parameters in the model estimated by the optimization algorithms and (ii) isolation of the mother function $H_{m}\left(s\right)$ from the fractional-order dispersion coefficients in the model.

**Figure 1:**
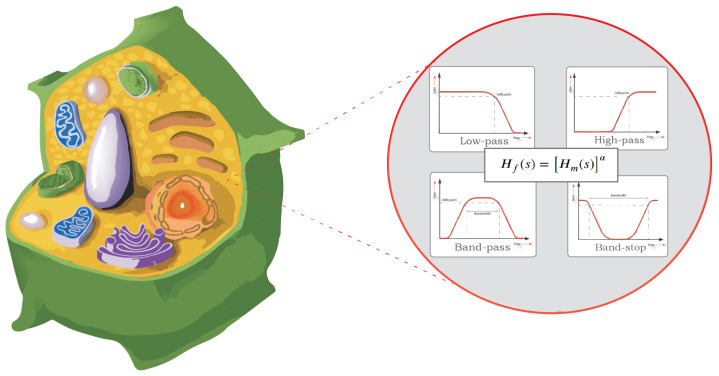
A 3D cross section of a typical plant cell structure and its modelling using power−law filters.

In this paper, bio-impedance models based on power-law filters are used and verified experimentally for fruit tissue representation. The impedance for eight fruit samples is measured using an electrochemical workstation, and the data is then fitted to different power-law filter types and combinations. The filters’ parameters are extracted using the water cycle algorithm (WCA). The error in fitting is calculated to find the best type of filter or combination of filters for each fruit as illustrated in Figure 1.

This paper is organized as follows. A brief description of power-law filters used in the proposed work is presented in Section 2. The methodology and problem definition is presented in Section 3. The experimental results and discussion are presented in Section 4, while the proposed work is concluded in Section 5.

## 2. Power-Law Filters

The power-law filters proposed in [32] have been employed in this work to validate the capability of power-law filters in modelling the behaviour of tissues.

### 2.1. Filters Based on First-Order Mother Functions

The transfer function of a first-order low pass (LP) based power-law filter with 0<α<1 is given by Equation (3a), where $H_{LP_{1}}\left(s\right)$ is the low-pass base function with gain defined as *K* and pole frequency $\omega_{o}$ as in (3b).

(3a) $$\begin{array}{cc} Z(s) & ={\left[H_{LP_{1}}\left(s\right)\right]}^{\alpha }, \end{array}$$

(3b) $$\begin{array}{cc} H_{LP_{1}}\left(s\right) & =\frac{K}{1+\frac{s}{\omega_{o}}}. \end{array}$$

The power-law high pass filter can be written as:

(4) $$H_{HP_{1}}\left(s\right)={\left[\frac{K\frac{s}{\omega_{o}}}{1+\frac{s}{\omega_{o}}}\right]}^{\alpha }.$$

The power-law first order all-pass filter transfer function is given by:

(5a) $$\begin{array}{cc} Z(s) & ={\left[H_{AP}\left(s\right)\right]}^{\alpha }, \end{array}$$

(5b) $$\begin{array}{cc} H_{AP}\left(s\right) & =\frac{K(1-\frac{s}{\omega_{o}})}{1+\frac{s}{\omega_{o}}}. \end{array}$$

### 2.2. Filters Based on Second-Order Mother Functions

The transfer function of the power-law second-order LP filter is given in Equation (6) with base function $H_{LP2}\left(s\right)$ (gain *K*, pole frequency $\omega_{o}$ and quality factor *Q*)

(6a) $$\begin{array}{cc} Z(s) & ={\left[H_{LP_{2}}\left(s\right)\right]}^{\alpha }, \end{array}$$

(6b) $$\begin{array}{cc} H_{LP_{2}}\left(s\right) & =\frac{K\omega_{o}^{2}}{s^{2}+\frac{\omega_{o}}{Q}s+\omega_{o}^{2}}. \end{array}$$

The corresponding expression for a second-order power-law HP filter is given by:

(7) $$H_{HP_{2}}\left(s\right)={\left[\frac{s^{2}}{s^{2}+\frac{\omega_{o}}{Q}s+\omega_{o}^{2}}\right]}^{\alpha }.$$

For a power-law band-pass filter, the transfer function is given in Equation (8) with base function $H_{BP}\left(s\right)$,

(8a) $$\begin{array}{cc} Z(s) & ={\left[H_{BP}\left(s\right)\right]}^{\alpha }, \end{array}$$

(8b) $$\begin{array}{cc} H_{BP}\left(s\right) & =\frac{K\frac{\omega_{o}}{Q}s}{s^{2}+\frac{\omega_{o}}{Q}s+\omega_{o}^{2}}. \end{array}$$

### 2.3. Power-Law Filter Sections

In [34], the double Cole model was visualized as a sum of power-law filter sections described by Equation (9). This technique provides flexibility in deciding the number of unknown parameters found through optimization techniques.

(9) $$Z_{n}\left(s\right)=1+{\left[H_{m_{1}}\left(s\right)\right]}^{\alpha }+{\left[H_{m_{2}}\left(s\right)\right]}^{\beta }.$$

Several combinations of power-law filter sections are studied such as the combination of two first-order power-law LP filters (see Equation (10)) referred to as (combination 1) later on,

(10) $$Z\left(s\right)={\left[H_{LP_{1}}\left(s\right)\right]}^{\alpha }+{\left[H_{LP_{1}}\left(s\right)\right]}^{\beta }.$$

or the combination (combination 2) of two second-order power-law LP filter sections (see Equation (11)) or the combination of a first-order LPF and APF, as given by Equation (12) (combination 3). Each filter has its gain *K*, pole frequency $\omega_{o}$ and the fractional-order powers (α,β).

(11) $$Z\left(s\right)={\left[H_{LP_{2}}\left(s\right)\right]}^{\alpha }+{\left[H_{LP_{2}}\left(s\right)\right]}^{\beta }.$$

(12) $$Z\left(s\right)={\left[H_{LP_{1}}\left(s\right)\right]}^{\alpha }+{\left[H_{AP}\left(s\right)\right]}^{\beta }.$$

A combination of a power-law first-order LPF and a second-order power-law BPF is given by Equation (13) (combination 4), and a combination of power-law BPF and APF is given by Equation (14) (combination 5).

(13) $$Z\left(s\right)={\left[H_{LP_{1}}\left(s\right)\right]}^{\alpha }+{\left[H_{BP}\left(s\right)\right]}^{\beta }.$$

(14) $$Z\left(s\right)={\left[H_{BP}\left(s\right)\right]}^{\alpha }+{\left[H_{AP}\left(s\right)\right]}^{\beta }.$$

## 3. Problem Definition

**Figure 2:**
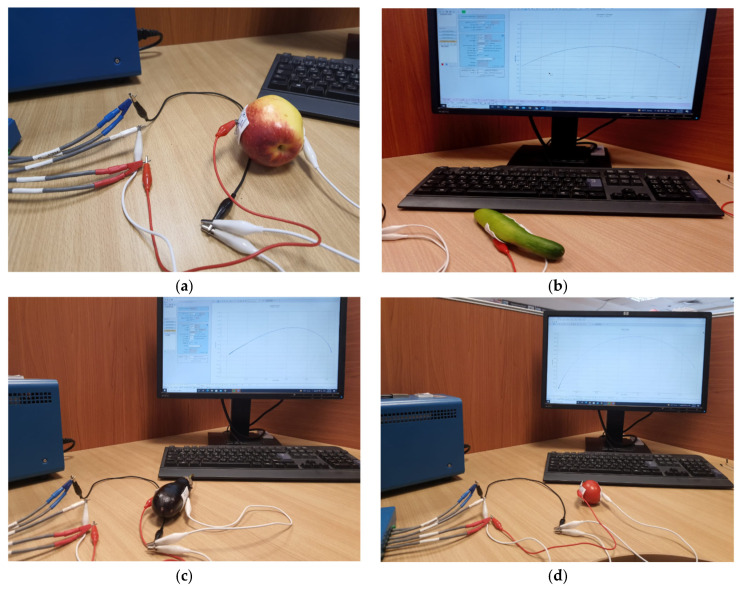
Experimental setup using the SP150 electrochemical station for measuring impedance data of four fruit samples: (**a**) apple; (**b**) cucumber; (**c**) eggplant; and (**d**) tomato.

Two samples of eight fresh fruit types (apple, cucumber, eggplant, kiwi, peach, plum, pear and tomato) are picked from a local grocery and employed in the experiment. The impedance of each fruit type is measured as in Figure 2 in the frequency range 10 Hz to 100 kHz with 80 points per decade at a room temperature of 25 ${}^{\circ }$C. Non-invasive Ag/AgCl electrodes are centred symmetrically around the fruit samples. A sinusoidal voltage of $V_{rms}$ = 20 mV is applied to each sample with no DC offset. The measured impedance is imported to Matlab as an input to the post-processing algorithm.

To extract the employed models parameters’ $\left(K_{i},\omega_{oi},Q_{i},\alpha ,\beta \right)$, WCA is used. Several factors affect the operation of the optimization algorithm such as the upper and lower boundaries, the number of search agents, the vector of optimized variables, the objective function and the number of iterations. These factors are described as follows:

1. The objective function (f(x)) used by the optimization algorithm is the minimization of the sum of the absolute error between the estimated impedance from the power-law filter and the measured one for each point in the frequency range represented as:
  (15) $$f\left(x\right)=min\sum_{i=1}^{n}|\frac{Z{\left(s_{i}\right)}_{model}-Z{\left(s_{i}\right)}_{measured}}{Z{\left(s_{i}\right)}_{measured}}|,$$
  where *x* is the vector of the optimized variables, model parameters of each filter type depending on the problem size, $Z{\left(s_{i}\right)}_{model}$ is the power-law filter impedance, $Z{\left(s_{i}\right)}_{measured}$ is the actual-measured impedance of the sample, while *n* is the total number of the measured points.
2. The number of iterations used in the optimization is 2500 iterations with 60 search agents and 50 independent runs through all the tested samples.
3. The search agents search for the best solution in a range defined between a lower (LB) and an upper (UB) boundary defined differently for each filter order. For first-order filters, LB = [α, *K*, $\omega_{o}$] = [0, 0, 0] and UB = [1, 1$\;\times \;10^{11}$, 1$\;\times \;10^{8}$], while for second-order filters LB = [α, *K*, $\omega_{o}$, *Q*] = [0, 0, 0, 0] and UB = [1, 1$\;\times \;10^{11}$, 1 $\;\times \;10^{8}$, 10].

## 4. Results and Discussion

The extracted parameters from using WCA are demonstrated in Table 1. The purpose is to find the most suitable power-law filter-based models for characterizing a given plant tissue. The Nyquist plot represents the experimental and fitted results for each fruit sample to demonstrate the effect on real and imaginary data along the frequency range. The Nyquist plot depends on tissues’ homogeneity, structure, ion concentration, water content and peel shape [13]. For the tested samples, cucumber, eggplant, peach and tomato have the most water content compared to other samples, with an average of 95% of the whole fruit size and very thin peel shape [37], where the cucumber contains the highest water content. The Nyquist plot of the four fruit samples showed lower measured impedance at low frequency compared to the other samples, with the lowest impedance obtained from the cucumber sample. Apple and pear samples have equivalent average water content up to 84% and are similar in peel structure, thus demonstrating high impedance response at low frequencies.

### 4.1. Models Based on Single Power-Law Filters

Table 2 shows the Nyquist plot for fitting results of three power-law filters (first-order LPF, second-order LPF and second-order BPF), also defined as single filters, described in Equations (3), (6) and (8), and the Nyquist plot of the actual measured impedance. Furthermore, the error between the employed filter’s response and the actual data is plotted for the applied frequency range to determine the most suitable model.

For the bio-impedance model based on power-law first-order LPF, the maximum error between the experimental and fitted impedance Nyquist plot reaches 7% for apple, plum, eggplant and tomato samples, 3.5% for cucumber, 5% for kiwi and peach, while it reaches more than 10% for pear. For the model based on power-law second-order LPF, the maximum error between the experimental and fitted impedance Nyquist plot reaches 6.5% for apple and tomato, 3.5% for cucumber, 7% for eggplant and plum, 5% for kiwi and peach, while it reaches 8% for pear. For the model based on power-law second-order BPF, the maximum error between the experimental and fitted impedance reaches 7% for apple, plum, eggplant and tomato samples, 3.5% for cucumber, 5% for Kiwi and peach, while it reaches more than 10% for pear.

Notice that the model based on the second-order BPF showed similar error results to that based on the first-order LPF in all fruit samples. The model based on the power-law second-order LPF showed a near similar response to the previously mentioned two models for kiwi and peach samples. The first-order and second-order HPF described in Equations (4) and (7) failed across the whole frequency range, confirming the capacitive nature of biological tissues resulting in LPF behaviour.

### 4.2. Models Based on Power-Law Filters Sections

Power-law filter sections, (also defined as double filters) were also studied. Table 3 shows the Nyquist plot for fitting results of the five combinations of power-law filters mentioned earlier. A combination of two first-order LPF sections (combination one) and described in Equation (10) results in a maximum error between experimental and fitted impedance Nyquist plot that reaches approx. 2% for apple, eggplant and plum, less than 1% for cucumber, 3% for kiwi, 4% for peach, 8% for pear and 7% for tomato. A combination of two second-order LPF sections (combination two) described in Equation (11) results in a maximum error between experimental and fitted impedance that reaches approx. 1% for apple, less than 1% for cucumber and eggplant, 3% for kiwi and peach, less than 2% for pear and tomato and 2.5% for plum. Combination three, described in Equation (12), results in a maximum error between experimental and fitted impedance that reaches approx. 7% for apple and tomato, 3.5% for cucumber, 8% for eggplant and plum, 5% for kiwi, 4.5% for peach and 12.5% for pear. Combination four (see Equation (13)) results in a maximum error of approx. 4% for apple and eggplant, 3.5% for cucumber and kiwi, 3% for peach, less than 3% for pear, 9% for plum and 7% for tomato. Finally, combination five described in Equation (14) results in a maximum error of approx. 7% for apple and tomato, 1.5% for cucumber, 6% for eggplant, 5% for kiwi, 4.5% for peach, 12% for pear and 8% for plum.

Power-law second-order LPF sections combination (Combination 2) provides the best results with the slightest maximum error in most of the samples, followed by the power-law first-order LPF sections combination (Combination 1). The combination of the power-law first-order LPF and the second-order BPF (Combination 4) follows combinations 1 and 2 as the third-best result, while the usage of the all-pass filter does not enhance the fitting problem.

In conclusion, the combination of two fractional-order power-law filter sections shows better fitting results compared to models based on single filters in most cases. The combination of two power-law second-order LPF sections (Combination 2) gives the lowest maximum error in this work.

**Table 2:** Using WCA optimisation, the Nyquist and error plot of the experimental and the fitted single filters.

	Nyquist	Error
Apple	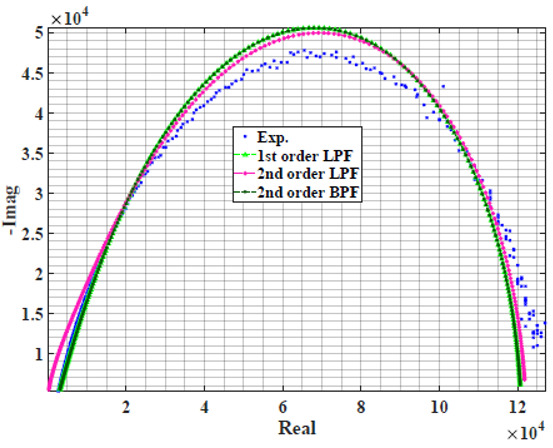	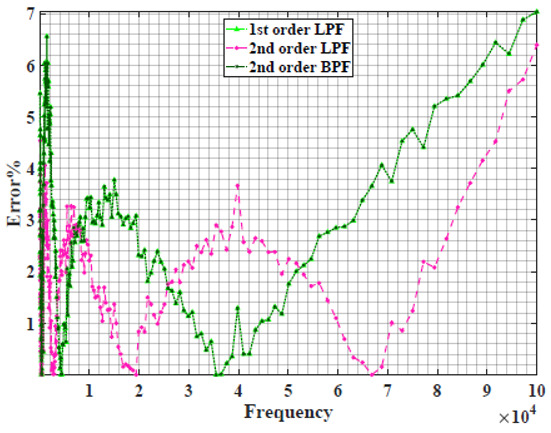
Cucumber	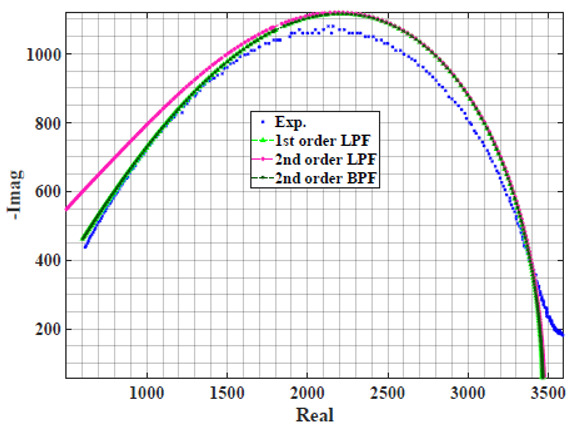	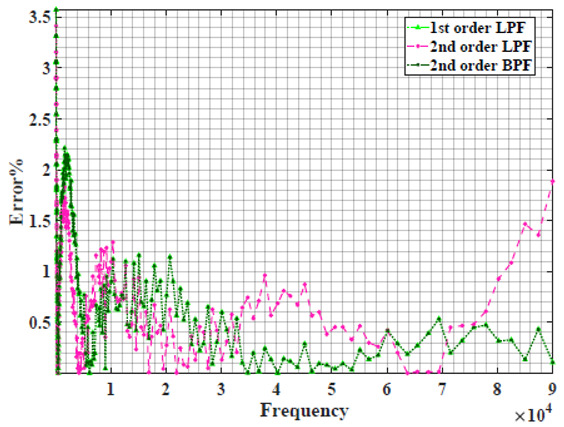
Eggplant	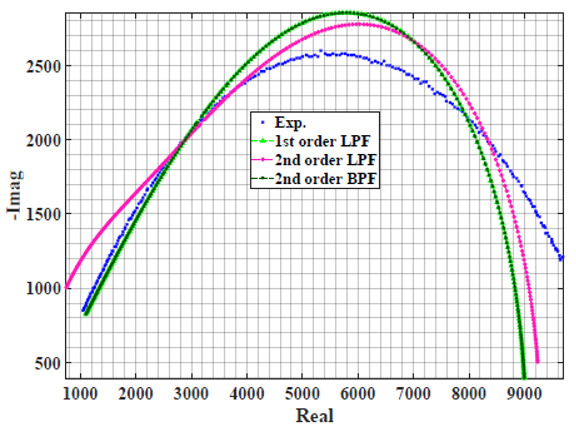	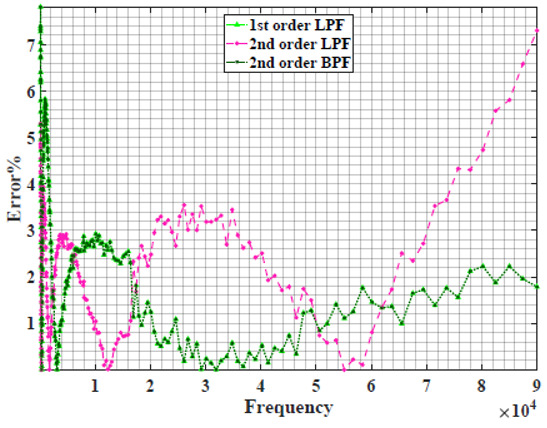
Kiwi	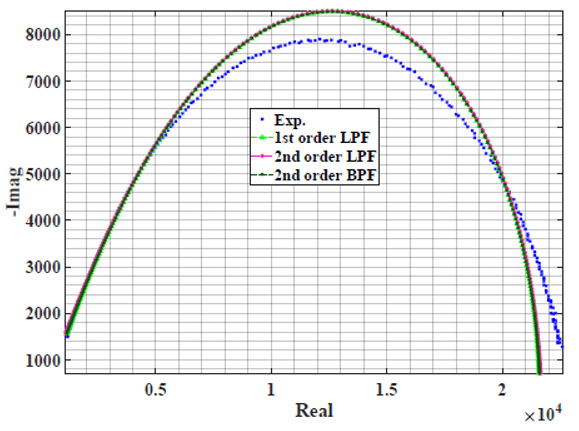	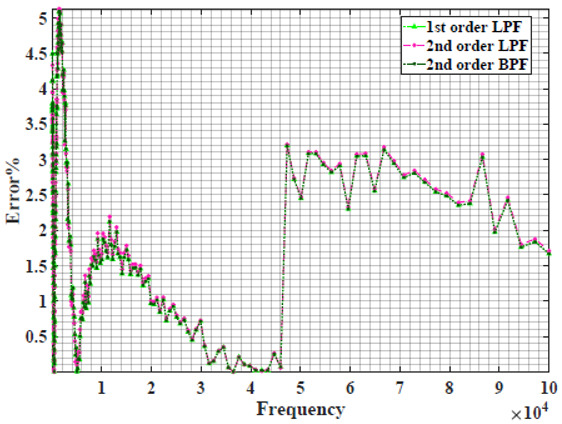
Peach	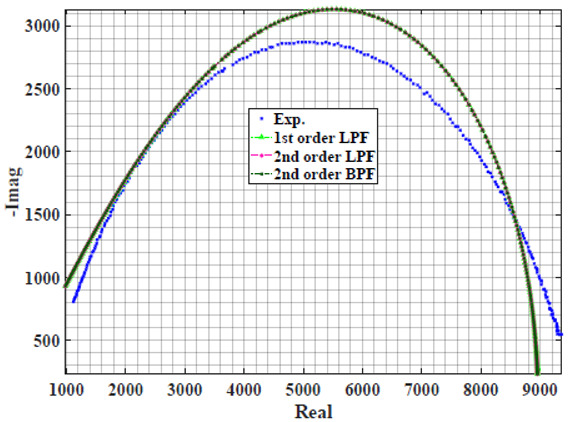	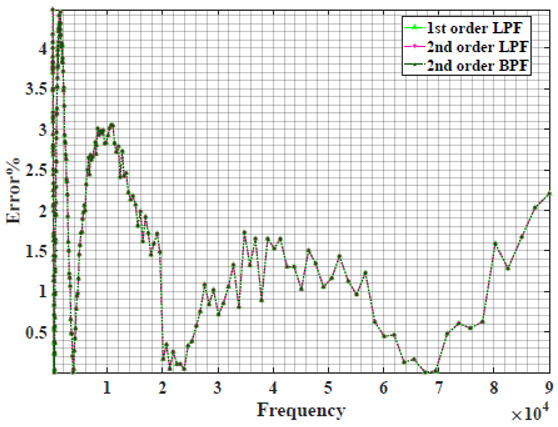
Pear	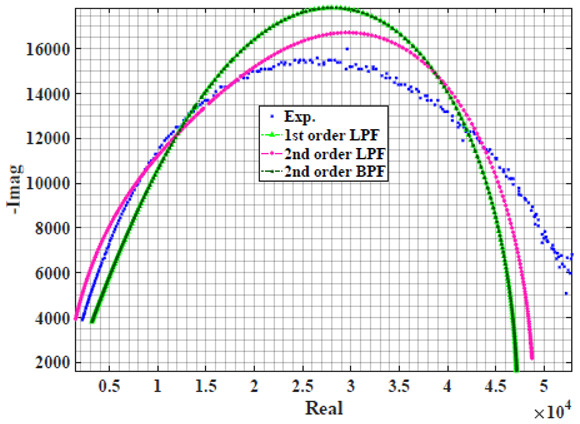	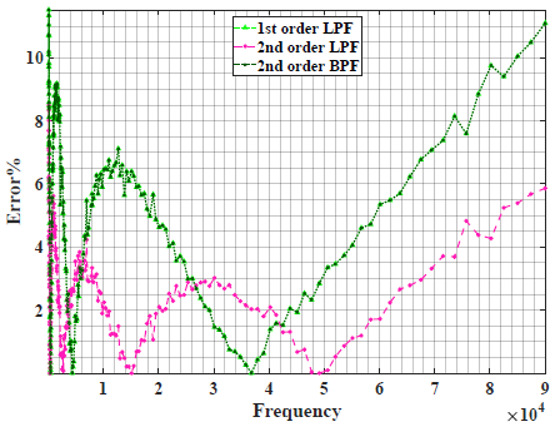
Plum	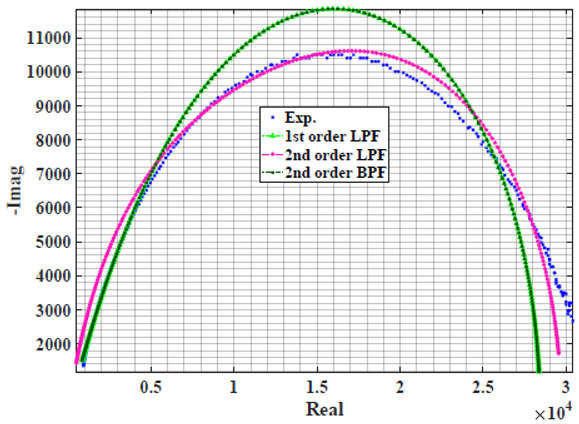	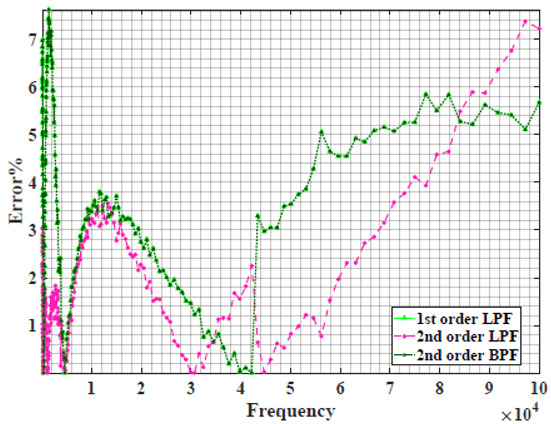
Tomato	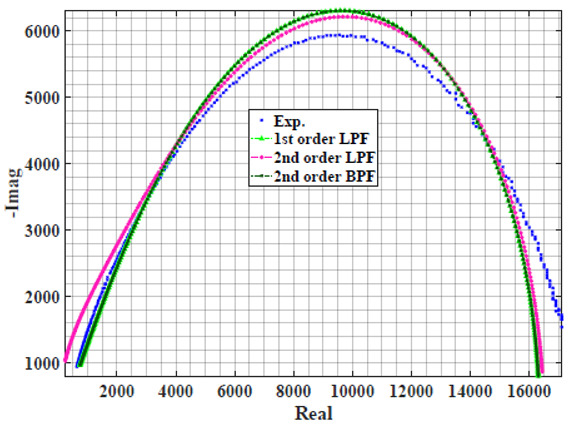	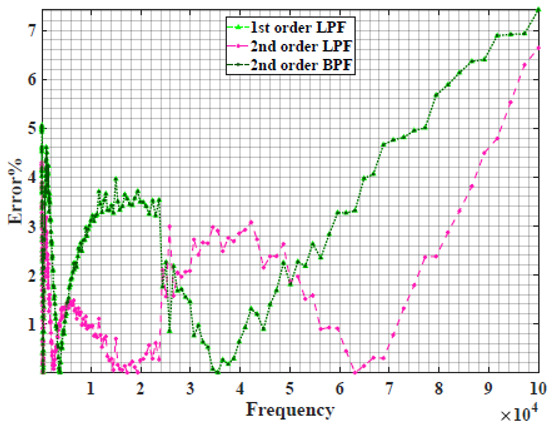

**Table 3:** Using WCA optimisation, the Nyquist and error plot of the experimental and the fitted double filters.

	Nyquist	Error
Apple	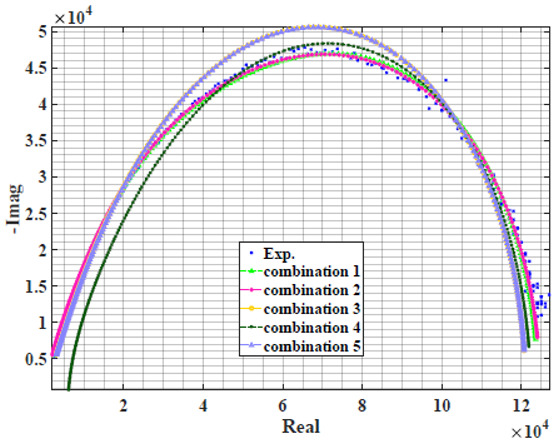	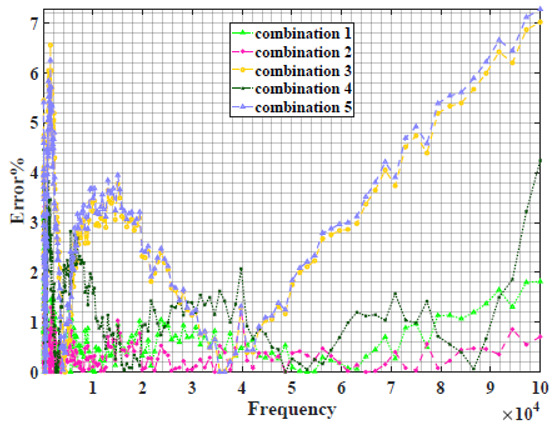
Cucumber	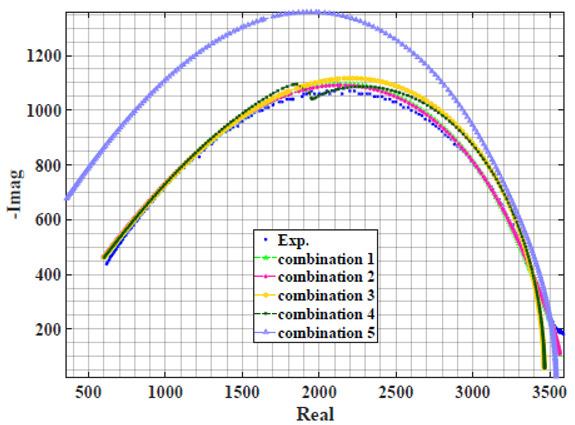	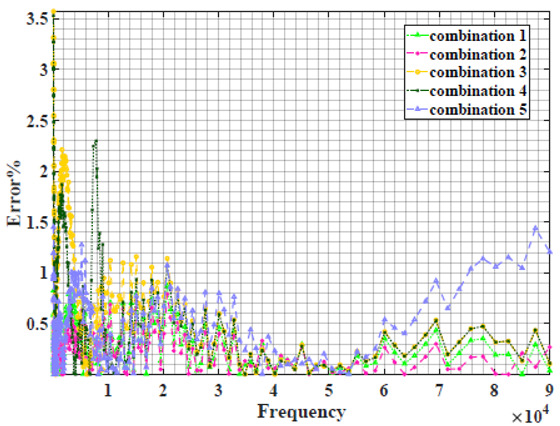
Eggplant	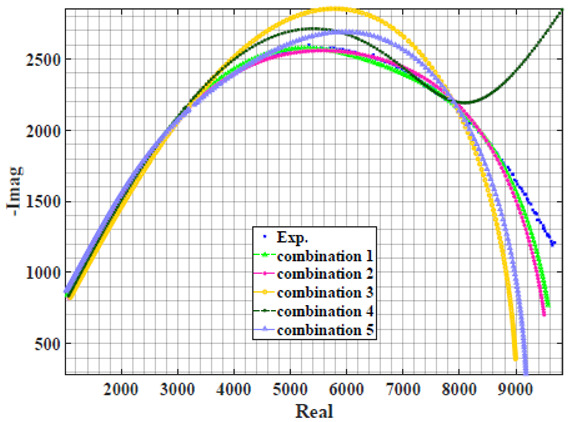	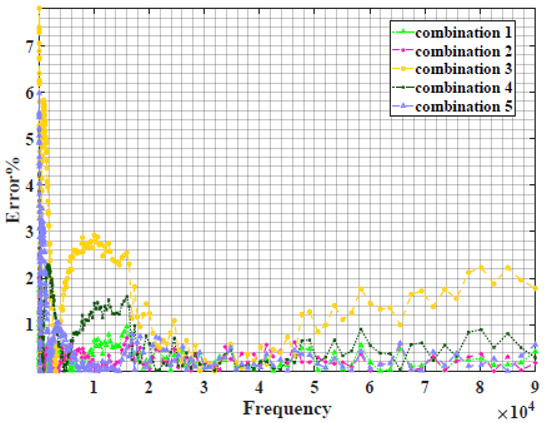
Kiwi	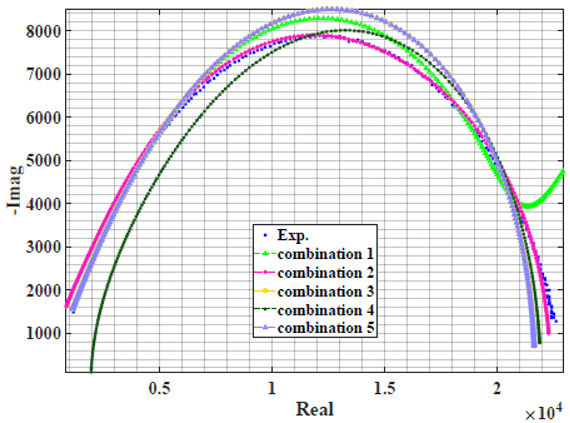	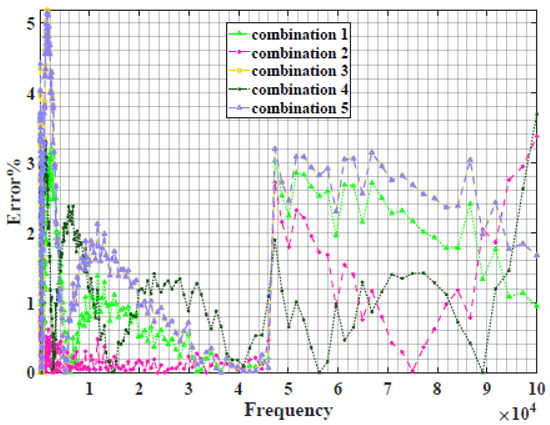
Peach	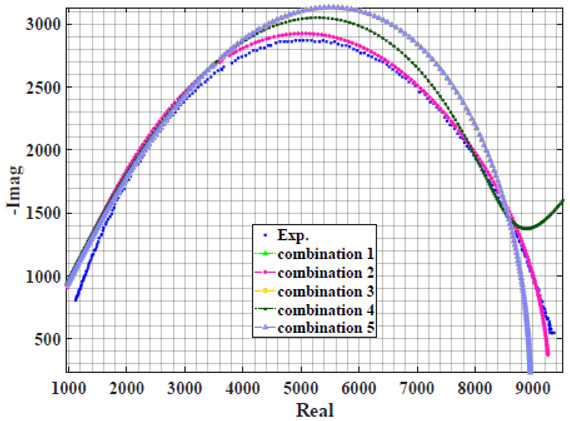	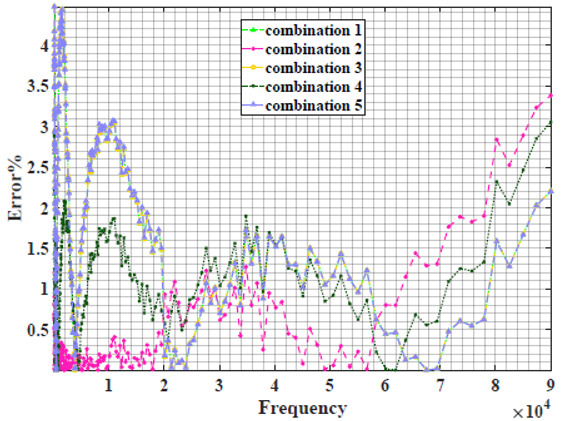
Pear	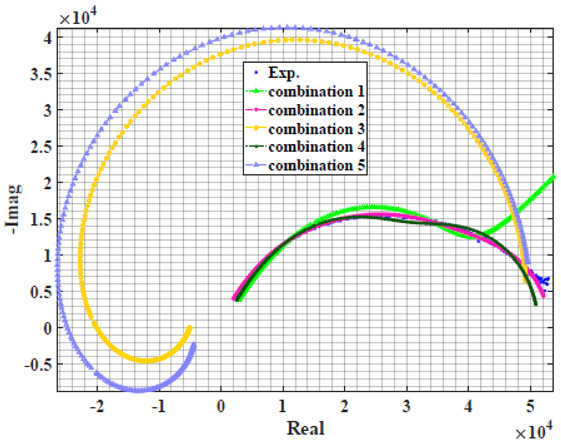	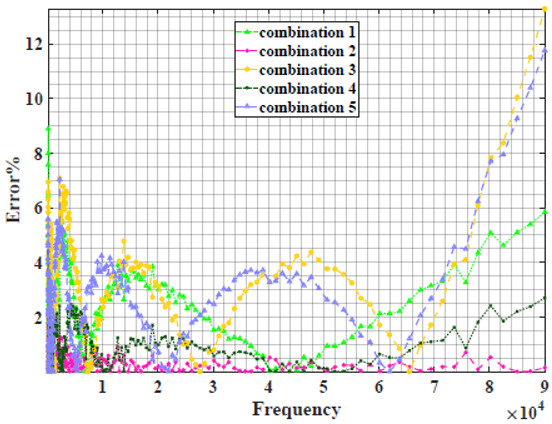
Plum	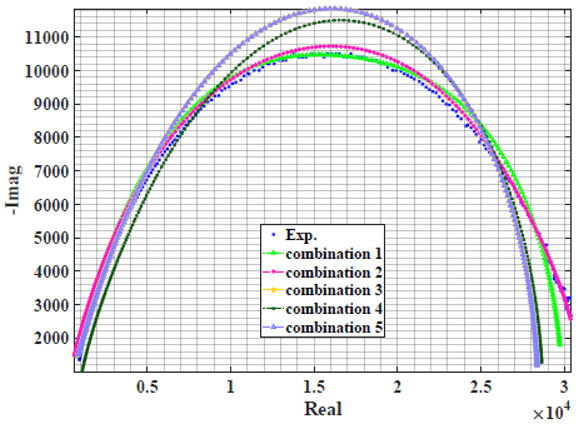	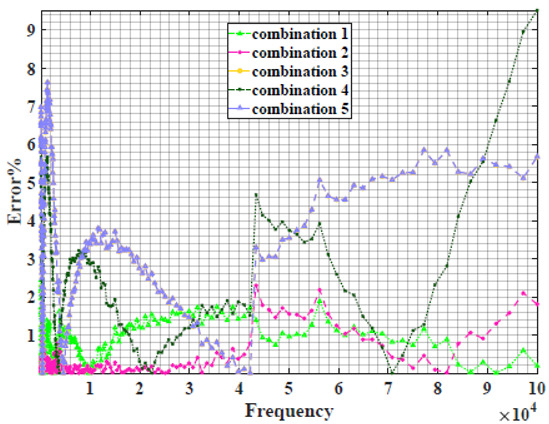
Tomato	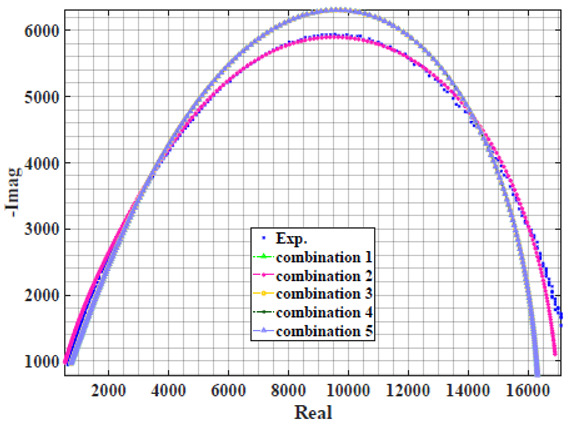	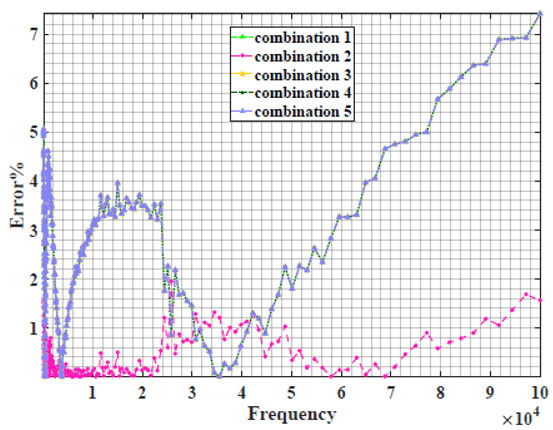

## 5. Conclusions

This paper proposes new bio-impedance models based on fractional-order power-law filters. The biological tissue is modelled as a power-law filter or a combination of power-law filter sections, thus aiding in capturing the magnitude and phase variations of the tissue response over a wide frequency range while isolating the non-integer power-law exponents from the underlying frequency-domain behaviour of all capacitive materials. The fractional-order power-law filters were applied in two forms: as a single filter section such as first-order and second-order LPF and second-order BPF and as double filter sections represented by five combinations of basic power-law filters. For models based on single filter sections, the power-law second-order LPF showed the lowest maximum error for all samples. For models based on double filter sections, the combination of two power-law second-order LPF sections showed the lowest maximum error for all samples. In general, the combination of more than one LP fractional-order power-law filter section, even if it is first-order or second-order, showed more satisfactory results for all tested samples.

## Figures and Tables

**Table 1:** Extracted parameters from the measured fruit samples using the WCA optimization technique.

		Parameters	$K_{1}$	$Q_{1}$	$\omega_{o_{1}}$	α	$K_{2}$	$Q_{2}$	$\omega_{o_{2}}$	β
Samples										
Single Filter	LP${}_{1}$	Apple	$2.65\times 10^{7}$	-	$8.48\times 10^{3}$	0.6846	-	-	-	-
		Cucumber	$2.14\times 10^{8}$	-	$1.57\times 10^{4}$	0.4248	-	-	-	-
		Eggplant	$3.92\times 10^{9}$	-	$5.89\times 10^{3}$	0.4122	-	-	-	-
		Kiwi	$1.43\times 10^{7}$	-	$1.16\times 10^{4}$	0.6058	-	-	-	-
		Peach	$1.18\times 10^{8}$	-	$1.18\times 10^{4}$	0.4896	-	-	-	-
		Pear	$1.97\times 10^{8}$	-	$1.04\times 10^{4}$	0.5634	-	-	-	-
		Plum	$3.48\times 10^{6}$	-	$1.03\times 10^{4}$	0.6807	-	-	-	-
		Tomato	$1.58\times 10^{7}$	-	$7.47\times 10^{3}$	0.5851	-	-	-	-
	LP${}_{2}$	Apple	$1.04\times 10^{8}$	$1.13\times 10^{-1}$	$6.34\times 10^{4}$	0.6344	-	-	-	-
		Cucumber	$4.36\times 10^{8}$	$1.15\times 10^{-1}$	$1.26\times 10^{5}$	0.4097	-	-	-	-
		Eggplant	$8.64\times 10^{10}$	$1.06\times 10^{-1}$	$3.89\times 10^{4}$	0.3628	-	-	-	-
		Kiwi	$1.46\times 10^{7}$	$2.64\times 10^{-2}$	$4.37\times 10^{5}$	0.6050	-	-	-	-
		Peach	$1.18\times 10^{8}$	$5.00\times 10^{-4}$	$2.37\times 10^{7}$	0.4896	-	-	-	-
		Pear	$9.98\times 10^{10}$	$1.84\times 10^{-1}$	$3.24\times 10^{4}$	0.4263	-	-	-	-
		Plum	$9.64\times 10^{10}$	$2.69\times 10^{-1}$	$1.62\times 10^{4}$	0.4066	-	-	-	-
		Tomato	$6.04\times 10^{7}$	$1.13\times 10^{-1}$	$5.63\times 10^{4}$	0.5419	-	-	-	-
	BP	Apple	$2.65\times 10^{7}$	$8.30\times 10^{-10}$	$7.04\times 10^{-6}$	0.6846	-	-	-	-
		Cucumber	$2.14\times 10^{8}$	$5.29\times 10^{-4}$	8.35	0.4248	-	-	-	-
		Eggplant	$3.92\times 10^{9}$	$2.71\times 10^{-3}$	16.02	0.4122	-	-	-	-
		Kiwi	$1.43\times 10^{7}$	$6.46\times 10^{-4}$	7.53	0.6058	-	-	-	-
		Peach	$1.18\times 10^{8}$	$8.09\times 10^{-4}$	9.59	0.4896	-	-	-	-
		Pear	$1.97\times 10^{8}$	$4.68\times 10^{-12}$	$4.84\times 10^{-8}$	0.5634	-	-	-	-
		Plum	$3.48\times 10^{6}$	$1.31\times 10^{-4}$	1.35	0.6807	-	-	-	-
		Tomato	$1.58\times 10^{7}$	$1.77\times 10^{-6}$	$1.32\times 10^{-2}$	0.5851	-	-	-	-
Double Filters	LP${}_{1}$ & LP${}_{1}$	Apple	$4.15\times 10^{6}$	-	$2.24\times 10^{4}$	0.6981	$1.36\times 10^{6}$	-	$5.98\times 10^{3}$	0.8015
		Cucumber	$1.92\times 10^{9}$	-	$1.70\times 10^{3}$	0.2987	$6.19\times 10^{7}$	-	$1.87\times 10^{4}$	0.4459
		Eggplant	$7.03\times 10^{8}$	-	$2.03\times 10^{3}$	0.4234	$1.45\times 10^{8}$	-	$1.50\times 10^{4}$	0.4431
		Kiwi	$2.24\times 10^{9}$	-	$3.27\times 10^{3}$	0.5400	$8.44\times 10^{6}$	-	$1.34\times 10^{4}$	0.6184
		Peach	$1.06\times 10^{5}$	-	$2.25\times 10^{4}$	0.7046	$1.18\times 10^{8}$	-	$1.18\times 10^{4}$	0.4896
		Pear	$1.21\times 10^{7}$	-	$1.84\times 10^{4}$	0.6374	$8.28\times 10^{3}$	-	$5.00\times 10^{4}$	0.4893
		Plum	$1.24\times 10^{4}$	-	$5.42\times 10^{3}$	0.9581	$9.51\times 10^{5}$	-	$2.05\times 10^{4}$	0.7095
		Tomato	$1.58\times 10^{7}$	-	$7.47\times 10^{3}$	0.0061	$3.57\times 10^{3}$	-	$9.99\times 10^{6}$	0.5851
	LP${}_{2}$ & LP${}_{2}$	Apple	$8.44\times 10^{10}$	0.41	$1.28\times 10^{5}$	0.3617	$3.28\times 10^{10}$	0.32	$1.30\times 10^{4}$	0.4803
		Cucumber	$5.86\times 10^{10}$	0.36	$3.20\times 10^{3}$	0.2479	$1.45\times 10^{8}$	0.033	$5.59\times 10^{6}$	0.4268
		Eggplant	$8.50\times 10^{10}$	0.42	$2.27\times 10^{4}$	0.3119	$5.00\times 10^{10}$	$2.25\times 10^{-4}$	$7.77\times 10^{6}$	0.3491
		Kiwi	$4.39\times 10^{4}$	0.31	$6.04\times 10^{4}$	0.8244	$3.89\times 10^{10}$	0.11	$9.43\times 10^{6}$	0.3812
		Peach	$9.99\times 10^{10}$	0.016	$1.88\times 10^{5}$	0.3298	$1.94\times 10^{10}$	0.44	$2.44\times 10^{4}$	0.3366
		Pear	$1.11\times 10^{10}$	0.049	$3.69\times 10^{4}$	0.4399	$9.97\times 10^{10}$	0.34	$3.11\times 10^{4}$	0.4024
		Plum	$3.21\times 10^{10}$	0.084	$1.17\times 10^{4}$	0.3535	$9.07\times 10^{10}$	0.36	$1.44\times 10^{4}$	0.3985
		Tomato	$5.30\times 10^{10}$	0.24	$3.85\times 10^{4}$	0.3418	$4.74\times 10^{10}$	0.35	$8.20\times 10^{3}$	0.3833
	LP${}_{1}$ & AP	Apple	$2.64\times 10^{7}$	-	$2.64\times 10^{7}$	0.6847	0	-	0.84	0.0310
		Cucumber	$2.14\times 10^{8}$	-	$1.57\times 10^{4}$	0.4248	0	-	$4.35\times 10^{-6}$	0.0087
		Eggplant	$3.92\times 10^{9}$	-	$5.89\times 10^{3}$	0.4122	0	-	$3.12\times 10^{4}$	0.4148
		Kiwi	$1.44\times 10^{7}$	-	$1.16\times 10^{4}$	0.6057	0	-	0.053	0.0028
		Peach	$1.18\times 10^{8}$	-	$1.18\times 10^{4}$	0.4896	0	-	$9.75\times 10^{6}$	0.0749
		Pear	$1.95\times 10^{8}$	-	$1.03\times 10^{4}$	0.5638	0	-	$9.31\times 10^{6}$	0.0003
		Plum	$3.48\times 10^{6}$	-	$1.03\times 10^{4}$	0.6807	0	-	$2.73\times 10^{5}$	0.0844
		Tomato	$1.55\times 10^{7}$	-	$7.48\times 10^{3}$	0.5858	0	-	$9.89\times 10^{6}$	0.0001
	LP${}_{1}$ & BP	Apple	$1.67\times 10^{8}$	-	$7.07\times 10^{3}$	0.6187	$7.75\times 10^{6}$	3.89	$9.43\times 10^{6}$	0.8543
		Cucumber	$2.21\times 10^{8}$	-	$1.49\times 10^{4}$	0.4241	$7.02\times 10^{4}$	7.95	$4.62\times 10^{4}$	0.4335
		Eggplant	$5.21\times 10^{8}$	-	$9.92\times 10^{3}$	0.4342	$9.49\times 10^{10}$	4.46	39.33	0.4003
		Kiwi	$1.18\times 10^{8}$	-	$9.45\times 10^{3}$	0.5376	$1.56\times 10^{4}$	0.35	$2.80\times 10^{6}$	0.9375
		Peach	$4.68\times 10^{7}$	-	$1.48\times 10^{4}$	0.5069	$5.35\times 10^{9}$	1.39	63.72	0.4339
		Pear	$1.16\times 10^{7}$	-	$2.67\times 10^{4}$	0.6352	$2.03\times 10^{4}$	$4.00\times 10^{-4}$	1.83	0.6393
		Plum	$1.15\times 10^{7}$	-	$8.91\times 10^{3}$	0.6311	$6.87\times 10^{4}$	8.92	$6.95\times 10^{6}$	0.8769
		Tomato	$1.58\times 10^{7}$	-	$7.47\times 10^{3}$	0.5851	$6.24\times 10^{9}$	1.04	$4.65\times 10^{6}$	0.9668
	BP & AP	Apple	$2.64\times 10^{7}$	-	$8.48\times 10^{3}$	0.6847	0	-	0.84	0.0003
		Cucumber	$2.14\times 10^{8}$	-	$1.57\times 10^{4}$	0.4248	0	-	$4.35\times 10^{-6}$	0.0087
		Eggplant	$3.92\times 10^{9}$	-	$5.89\times 10^{3}$	0.4122	0	-	$3.12\times 10^{4}$	0.4148
		Kiwi	$1.44\times 10^{7}$	-	$1.16\times 10^{4}$	0.6057	0	-	0.053	0.0002
		Peach	$1.18\times 10^{8}$	-	$1.18\times 10^{4}$	0.4896	0	-	$9.75\times 10^{6}$	0.0749
		Pear	$1.95\times 10^{8}$	-	$1.03\times 10^{4}$	0.5638	0	-	$9.99\times 10^{6}$	0.0003
		Plum	$3.48\times 10^{6}$	-	$1.03\times 10^{4}$	0.6807	0	-	$2.73\times 10^{5}$	0.0008
		Tomato	$1.55\times 10^{7}$	-	$7.48\times 10^{3}$	0.5858	0	-	$9.99\times 10^{6}$	0.0001
